# Development of a semi-quantitative food frequency questionnaire for use in United Arab Emirates and Kuwait based on local foods

**DOI:** 10.1186/1475-2891-4-18

**Published:** 2005-05-27

**Authors:** Mahshid Dehghan, Nawal Al Hamad, AfzalHussein Yusufali, Fathimunissa Nusrath, Salim Yusuf, Anwar T Merchant

**Affiliations:** 1 Hamilton General Hospital, Population Health Research Institute, 237 Barton, Street East, Hamilton, ON L8L 2X2, Canada; 2 Administration of Food and Nutrition, Ministry of Health, P.O. Box 42432, Shuwaikh 70655, Kuwait; 3 Cardiology & Cardio Thoracic Centre, Dubai Hospital, Department of Health & Medical Services, P.O.Box – 7272, Dubai, UAE

## Abstract

**Background:**

The Food Frequency Questionnaire (FFQ) is one of the most commonly used tools in epidemiologic studies to assess long-term nutritional exposure. The purpose of this study is to describe the development of a culture specific FFQ for Arab populations in the United Arab Emirates (UAE) and Kuwait.

**Methods:**

We interviewed samples of Arab populations over 18 years old in UAE and Kuwait assessing their dietary intakes using 24-hour dietary recall. Based on the most commonly reported foods and portion sizes, we constructed a food list with the units of measurement. The food list was converted to a Semi-Quantitative Food Frequency Questionnaire (SFFQ) format following the basic pattern of SFFQ using usual reported portions. The long SFFQ was field-tested, shortened and developed into the final SFFQ.

To estimate nutrients from mixed dishes we collected recipes of those mixed dishes that were commonly eaten, and estimated their nutritional content by using nutrient values of the ingredients that took into account method of preparation from the US Department of Agriculture's Food Composition Database.

**Results:**

The SFFQs consist of 153 and 152 items for UAE and Kuwait, respectively. The participants reported average intakes over the past year. On average the participants reported eating 3.4 servings/d of fruits and 3.1 servings/d of vegetables in UAE versus 2.8 servings/d of fruits and 3.2 servings/d of vegetables in Kuwait. Participants reported eating cereals 4.8 times/d in UAE and 5.3 times/d in Kuwait. The mean intake of dairy products was 2.2/d in UAE and 3.4 among Kuwaiti.

**Conclusion:**

We have developed SFFQs to measure diet in UAE and Kuwait that will serve the needs of public health researchers and clinicians and are currently validating those instruments.

## Background

The residents of the Arab countries in the Persian Gulf region have become more sedentary and have dramatically changed their diet over the last two decades. They consume more fat, meat, sugar, rice and wheat flour than before [1-4]. This has resulted in a rise in obesity, diabetes, and cardiovascular disease prevalence. There is a need to study the relation between diet and chronic diseases in this population, but there is no customized instrument to do so. The Food Frequency Questionnaire (FFQ) is the most commonly used method to assess diet in relation to chronic disease [5]. Based on a complete search of the literature (using Medline medical subject heading and text words) and personal communication with nutritionists in United Arab Emirates and Kuwait Universities, we decided to develop a SFFQ and accompanying food composition database for UAE and Kuwait.

## Subjects and methods

### Population

Our sample consisted of 326 apparently healthy persons of Arab origin, between the ages of 18 and 65 years, living in UAE and Kuwait in 2003–2004. Of these 126 provided information using the 24-hour dietary recall, and 200 participated in the testing of the long-FFQ in the SFFQ development.

### Development of the semi-quantitative food frequency questionnaire

To develop the SFFQ we went through the following steps: construction of a food list, definition of portion sizes, and assignment of frequency of consumption, pilot test of long-FFQ and assembling the selected food list into SFFQ.

### 24-hour Dietary Recall (DR)

Qualified nutritionists interviewed the participants. The nutritionists asked the participants what they ate in the previous 24-hours in direct chronological order from the first foods in the morning to the last foods before breakfast on the day of the interview. To standardize data collection, we prepared a manual of procedures for the interviewers. The interviewers used a food atlas modified from "Food portion sizes: A photographic Atlas" [6] containing coloured pictures of 8 different portion sizes of foods commonly eaten in UAE and Kuwait.

We constructed a long food list from information we obtained from the 24-hour dietary recalls, and supplemented it with foods derived from popular cookbooks, and suggestions of experienced local dieticians. We obtained the range of portion sizes from the 24-hour dietary recall and cookbooks. For items such as eggs we considered one of those items as a portion. For fruits such as banana or orange, a medium size was considered to be a "unit" of the fruit. For foods with non-unitary measures, such as grapes, we considered 1/2 cup to be one unit. For other foods one unit of a utensil that was mostly reported by respondents was assumed as one portion size. To get an estimate of the usual portion we used the mode of the portion size distribution for each food reported.

We formatted the SFFQ based on the pattern of the Harvard FFQ. We organized the main foods by the traditional food groups into seven food categories: 1. Milk, milk products and fats (including milk with different amounts of fat, Labnah, etc.), 2. vegetables (fresh or cooked), 3. fruit, 4. meat, eggs and meat products (including meat organs), 5. cereal and cereal product, 6. beverages 7. sweet and baked goods (Faaloodah, baklava, etc.) and nuts like almond, and hamoose.

To distinguish the fibre content of the diet and the quality of carbohydrates, we differentiated between the types of consumed cereal and cereal products (white or wholemeal bread, whole grain). In the last section of the FFQ we asked about type of cooking oils and fats and also, minerals including calcium, iron, zinc and multivitamins.

We used 9 categories to assess frequency of intake varying from "never or less than once a month to 6 or more times per day. For each food item, participants indicated their average frequency of consumption over the past year of a specified serving size by checking 1 of the 9 frequency categories. For foods that contain an extremely high amount of a particular nutrient but are used infrequently, such as liver, we re-categorized the options for frequency of intake. For instance, we used eliminated options of higher intake (once per day) but distinguished between never and less than once a month at the lower end. We will compute the daily intake based on the midpoint of the reported frequency category for each food item; for example we will take a response of "2–4/week" to be 3/7 or 0.43 times/day.

Although most fruits and vegetables are available year round in UAE and Kuwait, their intakes may vary depending upon cost and cultural preferences of foods by season. For this reason we designed specific questions for fruit and vegetable consumption by season. We determined the length of the season from local experts who used their experience.

We pilot tested the long-FFQ among 200 participants from the same populations (but not those who participated in the 24 hr DR). The objectives were to determine the completeness of the food list and to shorten the food list by deleting foods that were not commonly consumed. Based on the analysis of the pilot we completed the food list and created SFFQ.

#### Food Composition Database

We will use the food composition database to convert intakes of foods into nutrients. We constructed one nutrient database for UAE and Kuwait, as most foods are similar in both countries. We extracted the nutrient contents of the food items from Table SR17 of the USDA food database, which is available online, as the starting point to establish the database . From SR17 we chose those varieties of food items which are not very specific to a region and are more representative, for example; for apple we chose "apples, raw, with skin (NDB No:09003)" or for orange, we chose "oranges, raw – all commercial varieties (NDB No: 09200)". We used Statistical analyses SPSS 10.1 for windows (SPSS Inc, Chicago IL) for all analyses.

## Results

There were 326 participants with different occupations in Kuwait and UAE in our study. The populations were similar in UAE and Kuwait with respect to age, sex distribution, and BMI, but Kuwaiti women had higher education levels than those in UAE (Tables 1 and 2). No significant differences were noted between female and male respondents with respect to BMI (P = 0.4, 0.5 for UAE and Kuwait, respectively). However, in UAE, women had a higher mean BMI than men while in Kuwait men were heavier than women.

**Table 1:** Demographic characteristics of UAE participants

	**Men (N = 35)**	**Women (N = 91)**	**Overall (N = 126)**
Age	43.0 ± 11	34.3 ± 10	37.0 ± 11
BMI (kg/m^2^)	27.4 ± 4	28.7 ± 7	28.3 ± 7
**Education**			
None	22.7%	10%	13%
Primary school	9.0%	8%	9%
Secondary school	23%	30%	31%
Trade School	32%	3%	2%
University	23%	49%	45%
**Income (Dirham)**			
<5000	12.5%	9%	11%
5001–10000	12.5%	63%	60%
10001–15000	19%	24%	22%
>15000	12.5%	4%	6%

**Table 2:** Demographic characteristics of Kuwaiti participants

	**Men (N = 56)**	**Women (N = 145)**	**Overall (N = 201)**
Age	45.6 ± 14.0	37.3 ± 12	39.6 ± 13.3
BMI (kg/m^2^)	26.6 ± 7	25.9 ± 5	26.0 ± 6
**Education**			
None	0%	0%	0%
Primary school	0%	5%	4%
Secondary school	32%	18%	21%
Trade School	5%	4%	4%
University	64%	72%	70%
**Income**			
<500	68%	31%	61%
501–1000	32%	54%	36%
>1000	0%	15%	3%

### Daily food intake

The estimated daily intakes of seven major food groups among Kuwaiti and UAE men and women are shown in Tables 3 and 4. On average the participants in UAE reported eating 3.4 servings/d of fruits and 3.1 servings/d of vegetables versus 2.8 servings/d of fruits and 3.2 servings/d of vegetables in Kuwait. Cereals are an important staple in the diet of both countries and the participants reported eating cereals 4.8 times/d in UAE and 5.3 times/d in Kuwait. All participants reported consuming cereals at least once per day. Meat was consumed nearly two times/day in both countries and among the meat group, poultry was consumed more often than red meat or fish. The mean intake of dairy products was 2.2/day in UAE and 3.4/day in Kuwait.

**Table 3:** Average daily intake of main foods estimated by long-FFQ reported by UAE participants (Men = 24, Women = 76)

**Sex**	**Foods**	**Min**	**Max**	**Mean**	**Std. Deviation**
Women	Fruits	0.2	8.5	3.0	1.7
	Vegetables	0.4	7.9	3.0	1.5
	Dairy products	0.0	5.2	2.1	1.0
	Meat	0.6	4.2	1.7	0.7
	Cereals and cereal products	0.7	10.7	4.7	2.2
	Beverages	0.0	9.5	3.4	2.1
	Baked goods	0.0	3.4	0.5	0.5
Men	Fruits	1.8	8.8	4.8	2.0
	Vegetables	0.3	6.6	3.7	1.6
	Dairy products	0.4	7.0	2.5	1.3
	Meat	0.6	4.4	2.3	0.9
	Cereals and cereal products	1.5	6.6	5.0	1.2
	Beverages	1.3	8.5	5.0	1.9
	Baked goods	0.0	2.1	0.8	0.6
Overall	Fruits	0.2	8.8	3.4	1.9
	Vegetables	0.2	7.9	3.1	1.6
	Dairy products	0.2	7.1	2.2	1.1
	Meat	0.6	4.4	1.9	0.8
	Cereals and cereal products	0.7	10.8	4.8	2.0
	Beverages	0.0	9.5	3.8	2.2
	Baked goods	0.0	3.4	0.6	0.5

**Table 4:** Average daily intake of main foods estimated by long-FFQ reported by Kuwaiti participants (Men = 22, Women = 78)

**Sex**	**Foods**	**Min**	**Max**	**Mean**	**Std. Deviation**
Women	Fruits	0.4	8.1	2.8	1.7
	Vegetables	0.0	10.5	3.3	2.2
	Dairy products	0.0	9.7	3.3	2.3
	Meat	0.2	9.9	1.8	1.3
	Cereals and cereal products	0.7	20.1	4.9	3.2
	Beverages	0.0	11.1	2.9	2.3
	Baked goods	0.0	5.0	1.0	1.0
Men	Fruits	0.5	7.3	3.1	1.8
	Vegetables	0.3	6.5	3.0	1.9
	Dairy products	0.4	6.6	3.5	1.7
	Meat	0.4	6.6	2.5	1.6
	Cereals and cereal products	2.3	12.8	6.5	2.4
	Beverages	0.0	9.3	4.0	2.7
	Baked goods	0.0	5.7	1.2	1.5
Overall	Fruits	0.4	8	2.8	1.7
	Vegetables	0.00	10.5	3.2	2.1
	Dairy products	0.00	9.7	3.4	2.3
	Meat	0.2	9.9	1.9	1.4
	Cereals and cereal products	0.7	20.1	5.3	3.1
	Beverages	0.0	5.7	1.0	1.1
	Baked goods	0.0	11.0	3.1	2.4

### Frequency of consumed food

Tables 6 and 7 show the frequency of consumption of some foods in UAE and Kuwait. About 32% of respondents in UAE and 26% of Kuwaiti respondents reported that they had at least one glass of milk daily on average in the past year. Sixty-eight percent of UAE participants and 48% of Kuwaiti participants reported consuming rice at least once per day. 67% and 51% of people (UAE and Kuwaiti respectively) ate an egg at least 2 times/week. Overall in UAE, 86% of participants did not eat chicken with skin while in Kuwait 42% of participants did not eat chicken with skin. Among fruits, apples, oranges and bananas were consumed very frequently.

### Nutrient database

To convert local mixed dishes to nutrients, we created a new nutrient database appropriate for local foods (Table 5). Therefore, a local nutritionist collected 2 recipes from each low, middle and high-income family. We supplemented these recipes from the "Food composition: Kuwaiti composite dishes" [7] and other popular cookbooks. The average amount of ingredients from those recipes was used to create a base recipe for the nutrient database. We matched ingredients of the recipes with the appropriate food items in the USDA database to obtain nutrient content, taking cooking method into account. The SFFQ for UAE is in Appendix I and the SFFQ for Kuwait is in Appendix II.

**Table 5:** Nutrient composition per serving (100 g) of some commonly eaten foods in UAE and Kuwait

								**Vitamins (mg)**							
**Food**	**Total calories (Kcal)**	**CHO (g)**	**Fat (g)**	**Protein (g)**	**Fiber (g)**	**A (RE,μ) (α)**	**Folate (μ)**	**B1**	**B6**	**B12**	**C**	**E**	**Ca (mg)**	**Phosphorus (mg)**	**Iron (mg)**
**Mixed dish**															
Kofta	173	3.1	11.1	15.0	0.6	63.1	14.1	0.09	0.26	1.7	12.6	0.1	23.7	128	1.6
Qouzi	206	15.0	11.2	10.5	0.6	5.1	36.7	0.1	0.1	0.9	2.3	0.3	18.0	90.3	1.4
Marga Laham	95	7.1	4.0	7.5	0.8	19.0	4.6	0.07	0.2	0.7	7.0	0.04	7.1	65.4	0.8
Jereesh	226	29.7	7.0	12.9	5.2	13.9	22.7	0.2	0.2	0.7	3.5	0.4	21.1	252	2.1
**Sweets**															
Balalett	210	35	4.7	6.6	0.1	Trace	10	0.01	0.06	Trace	Trace	0.5	28.5	105	1.5
**Elba**	264	30.6	12.6	11.3	Trace	206	25	0.1	0.1	1.3	1.1	0.6	522	356	2.1

## Discussion

In this paper, we have described the development of a semi-quantitative FFQ and food composition database for the Arab population in UAE and Kuwait. The goals of diet assessment in epidemiologic studies are to obtain a measure of usual rather than current diet, and rank people by intake, in contrast to clinical settings where current absolute intake is more important. The FFQ has been developed with these purposes in mind and has become the standard method to collect dietary data in studies of chronic disease all over the world. We opted to use a semi-quantitative FFQ, which estimated food intake in categories rather than the exact frequency, because it has been shown that there is minimal loss of information in estimating nutrient intakes using food intake categories [8]. We also asked the participants about intakes of pre-specified portion sizes rather than asking them to estimate their regular portion size. Correlations for nutrient intake calculated using the FFQ with and without taking portion sizes into account were over 0.9 [5]. The advantage of using categories to estimate food intake and pre-specified portion sizes is that the SFFQ becomes easier to administer, and likely, more reliable. We did not attempt to make a comprehensive list of foods to include in the SFFQ. Rather, we kept items in the SFFQ if they were nutrient rich, consumed frequently and discriminated intakes between individuals. The other criterion we considered together with the nutrient content (including caloric value) was the presence of other substances of interest, for instance, caffeine. Most FFQs have between 100–150 items [9] and our SFFQ has 153 (UAE) and 152 (Kuwait) items. Increasing the number of items in the FFQ has been shown to increase over-reporting [10].

To estimate nutrient intake from SFFQ, there is a need for a food composition table listing the average nutrient content of foods contained in the SFFQ. To obtain nutrient intake we multiplied the average nutrient content of a specified portion of food listed in the food composition table by the average frequency of intake reported in the SFFQ. The food composition table can be a substantial source of variation in the estimation of nutrients using the SFFQ. As no nutritional database has ever been gathered in UAE or Kuwait, we used the US Department of Agriculture nutrient database as our standard to estimate nutrient content. The advantages of this approach are: First, the USDA food composition database is probably the most comprehensive in the world. For example, there are 26 categories of spinach including different types of spinach, raw spinach, and spinach cooked in a variety of ways [11], allowing us to choose the most appropriate one. Second, the nutrient estimation assays have been done in a standardized manner. Third, it has the largest number of nutrients reported. Fourth, the USDA food composition database is continually updated. Last, UAE and Kuwait import foods from all around the world and a mixture of food items from different regions are available in any market. For mixed dishes that were not listed in the USDA database, we calculated nutrient intake by analyzing recipes. Moreover, there are nearly 150 food composition tables in use around the world and their values are primarily based on USDA [12-14], and even European countries include nutrient information from USDA in their food composition tables [15,16]. Finally, similar approaches have been taken by other investigators in Israel, [17] and Costa Rica [18].

A limitation of this study is that the age groups represented in the UAE and Kuwait sample are mostly <50 years for both males and females. Thus, the overall impression in dietary habits is biased towards this younger group. For example, the consumption of rice as well as dates might be underestimated. The way to make it more accurate is of course to repeat it (validate). Another limitation of this study is that most participants from both countries were women and some foods, which men may eat, may be underestimated. However, a nutritionist with experience in those countries reviewed the food lists to ensure their completeness.

## Conclusion

The validated questionnaire and food composition database will not only be useful tools for our own study, but they will also be assets that other researchers in the region can use or adapt to suit their needs. We have enclosed two SFFQs in this article so other researchers in the field of public health can use this comprehensive SFFQ. We are currently evaluating SFFQs and the validated SFFQ will be available online for all public health researchers in the region.

## List of Abbreviations

SFFQ Semi-Quantitative Food Frequency Questionnaire

24 hr DR 24 hour Dietary Recall

UAE United Arab Emirates

PURE Prospective Urban and Rural Epidemiologic

SPSS Statistical Packages for Social Sciences

## Authors' contributions

MD Participated in design of study, coordinated and performed statistical analysis, drafted the manuscript

AM Participated in design of study, performed statistical analysis, helped to draft the manuscript

NH Coordinated study in Kuwait, helped to draft the manuscript

AY Facilitated data collection in UAE, helped to draft the manuscript

FN Coordinated study in UAE

SY Participated in design of study, helped to draft the manuscript

All authors read and approved the final manuscript.

## References

[B1] Alawadi F, Amine EK (1989). Overweight and Obesity in Kuwait. Journal of the Royal Society of Health.

[B2] Musaiger AO (1985). Nutrition Situation in the Arabian Gulf Countries. Journal of the Royal Society of Health.

[B3] Musaiger AO, Sungpuag P (1985). Composition of Mixed Dishes Commonly Consumed in the Arabian Gulf States. Ecology of Food and Nutrition.

[B4] Musaiger AO (1993). Sociocultural and Economic-Factors Affecting Food-Consumption Patterns in the Arab Countries. Journal of the Royal Society of Health.

[B5] Willett W, Willett W (1998). Food Frequency Methods. Nutritional Epidemiology.

[B6] M N, M A, J M (1997). A photographic Atlas of Food Portion Sizes.

[B7] Sawaya (1997). food composition: Kuwaiti composite dishes.

[B8] Willett W, Lenart E, Willett W (1998). Reproducibility and Validity of Food-Frequency Questionnaires. Nutritional Epidemiology.

[B9] W W (1994). Future directions in the development of food-frequecny questionnaires. Am J Clin Nutr.

[B10] P P, AM H, E H (1988). Reproducibility and validity of dietary assessment instruments: II. A qualitative food frequency questionnaire. Am J Epidemiol.

[B11] (2004). USDA National Nutrient Database for Standard Reference Release 17. http://www.nal.usda.gov/fnic/foodcomp.

[B12] Garcia V, Rona RJ, Chinn S (2004). Effect of the choice of food composition table on nutrient estimates: a comparison between the British and American (Chilean) tables
1. Public Health Nutrition.

[B13] WM R, AT P, SP M, JC K (1991). Compiling Data for Food Composition Data Bases.

[B14] W W Nutrition Epidemiology.

[B15] Hakala P, Knuts LR, Vuorinen A, Hammar N, Becker W (2003). Comparison of nutrient intake data calculated on the basis of two different databases. Results and experiences from a Swedish-Finnish study
14. European Journal of Clinical Nutrition.

[B16] Deharveng G, Charrondiere UR, Slimani N, Southgate DAT, Riboli E (1999). Comparison of nutrients in the food composition tables available in the nine European countries participating in EPIC
1. European Journal of Clinical Nutrition.

[B17] Shai I, Vardi H, Shahar DR, Azrad AB, Fraser D (2003). Adaptation of international nutrition databases and data-entry system tools to a specific population. Public Health Nutrition.

[B18] H C (2004).

